# Different prognostic impact of glucose uptake in visceral adipose tissue according to sex in patients with colorectal cancer

**DOI:** 10.1038/s41598-021-01086-9

**Published:** 2021-11-03

**Authors:** Jae-Hoon Lee, Soyoung Kim, Hye Sun Lee, Eun Jung Park, Seung Hyuk Baik, Tae Joo Jeon, Kang Young Lee, Young Hoon Ryu, Jeonghyun Kang

**Affiliations:** 1 Department of Nuclear Medicine, Gangnam Severance Hospital, Yonsei University College of Medicine, Seoul, Republic of Korea; 2 Biostatistics Collaboration Unit, Yonsei University College of Medicine, Seoul, Republic of Korea; 3 Department of Surgery, Gangnam Severance Hospital, Yonsei University College of Medicine, 211 Eonju-ro, Gangnam-gu, Seoul, 06273 Republic of Korea; 4 Department of Surgery, Severance Hospital, Yonsei University College of Medicine, Seoul, Republic of Korea

**Keywords:** Cancer, Colon cancer, Rectal cancer

## Abstract

The purpose of this study was to investigate whether sex differences in visceral fat volume and glucose uptake measured by positron emission tomography/computed tomography (PET/CT) in abdominal visceral fat can stratify overall survival (OS) in patients with colorectal cancer (CRC). We retrospectively enrolled 293 patients diagnosed with CRC who underwent PET/CT before surgical resection. Fluorodeoxyglucose uptake of visceral adipose tissue (VAT-SUV) and subcutaneous adiposity tissue (SAT-SUV) were measured using PET/CT. The relative VAT (rVAT) was defined as the visceral fat volume normalized to the total volume of fat (VAT plus SAT). We defined sex-specific cutoff values for VAT-SUV, SAT-SUV, and rVAT. Univariate and multivariate analyses using Cox proportional hazard regression analysis were performed to identify the independent prognostic factors. The study population comprised 181 men and 112 women. The rVAT (0.40 vs. 0.29, *p* < 0.001) and VAT-SUV (0.55 vs. 0.48, *p* = 0.007) were significantly greater in men than in women. High rVAT (than low rVAT) and high VAT-SUV (than low VAT-SUV) showed a worse prognosis in male and female patients, respectively. Multivariate analysis indicated that the combination of rVAT and VAT-SUV was an independent prognostic factor for predicting OS in both male and female patients. The combination of rVAT and VAT-SUV could differentiate the patients with the best survival outcome from the other three individual groups in female patients, but not in males. Glucose uptake and relative volume of visceral fat may provide a new risk stratification for patients with CRC, especially female patients.

## Introduction

Recently, the role of body composition has gained significance in the field of oncology^1,2^. The main reason for this is that structured body composition measurements have been shown to provide a more accurate prediction of patients' nutritional, metabolic, and even surgical outcomes or prognosis compared to traditional anthropometric data such as body mass index^3,4^. Visceral and subcutaneous adipose tissues have been most commonly evaluated, and previous studies have mainly used volumetric quantification of adiposity measured using computed tomography (CT)^2,3^. It is well known that body fat distribution differs by sex, and men tend to have higher visceral fat, whereas women tend to have more subcutaneous fat^5^. This observation was considered when previous investigators defined the cutoff value for visceral and subcutaneous fat, and the criteria for men and women were set differently^6^.

Metabolic activity in a fat compartment was recently shown to have clinical significance in various types of cancer, including colorectal cancer (CRC)^7–10^. Most studies evaluating the metabolic activity of visceral fat have not considered sex differences. Whereas, Nguyen et al. revealed that sex differences in relative visceral fat volume and glycolytic gene expression could stratify patients' overall survival in more detail in women, but not in men, in patients with clear cell renal cell carcinoma^11^.

However, to the best of our knowledge, the prognostic significance of glucose uptake and the volume of visceral fat based on sex has been rarely investigated in patients with CRC. Thus, the purpose of this study was to investigate the clinical impact of visceral fat glucose uptake and the relative volume of visceral fat measured on [^18^F]fluorodeoxyglucose (FDG) positron emission tomography/computed tomography (PET/CT) and hypothesized that the association between visceral adipose tissue (VAT)-derived volume and metabolic activity may be sex-dependent and linked with survival in patients with CRC.

## Methods

### Subjects

A total of 293 patients who underwent preoperative PET/CT examinations followed by curative surgical resection of stage I–IV CRCs between December 2007 and April 2014 were included in this study. The inclusion criteria were as follows: (1) histopathologically confirmed CRC; (2) availability of PET/CT data from one designated PET/CT scanner; and (3) evaluation of surgery with curative intent, including resection of distant metastasis. Exclusion criteria were as follows: (1) those who underwent PET/CT scans more than one month prior to surgery; (2) those with a history of hereditary nonpolyposis CRC, ulcerative colitis, or Crohn's disease; (3) FDG uptake in the tumor or target organ was not measurable.

The study protocol adhered to the ethical standards of the institutional and/or national research committees and 1964 Helsinki Declaration and its later amendments. The institutional review board of the Gangnam Severance Hospital, Yonsei University College of Medicine, approved this study and waived the requirement for written informed consent owing to the retrospective study design.

### Image acquisition and processing

[^18^F] PET/CT scans were performed on a hybrid PET/CT scanner (Biograph TruePoint 40, Siemens Healthcare Solutions USA, Inc., Knoxville, TN, USA) 1 h after the intravenous administration of FDG (5.5 MBq/kg). All patients fasted for at least 8 h and were confirmed to have blood glucose levels below 180 mg/dL before the intravenous administration of FDG. A low-dose CT scan for attenuation correction was first obtained from the skull base to the proximal thigh without contrast enhancement using automatic dose modulation (120 kVp, 40 mAs, and 5 mm slice thickness). PET images were then acquired from the skull base to the proximal thigh for 3 min per bed position in three-dimensional mode. Images were reconstructed onto a 168 × 168 matrix using ordered subset expectation maximization with attenuation using two iterations and 21 subsets.

### Image analysis

PET/CT images were reviewed by two experienced nuclear medicine physicians using the LIFEx package (version 6.3; http://www.lifexsoft.org^12^) without knowledge of clinical outcomes.

First, FDG uptake in primary colorectal cancers was measured. A region of interest (ROI) was manually drawn around the primary tumor, taking care to avoid physiologic FDG uptake, especially in the urinary bladder and both urinary tracts. The maximum standardized uptake value (SUV) was measured. SUV was calculated as [decay-corrected activity (kBq) per tissue volume (mL)]/ (injected FDG activity [kBq] per body mass [g]).

**Figure 1:**
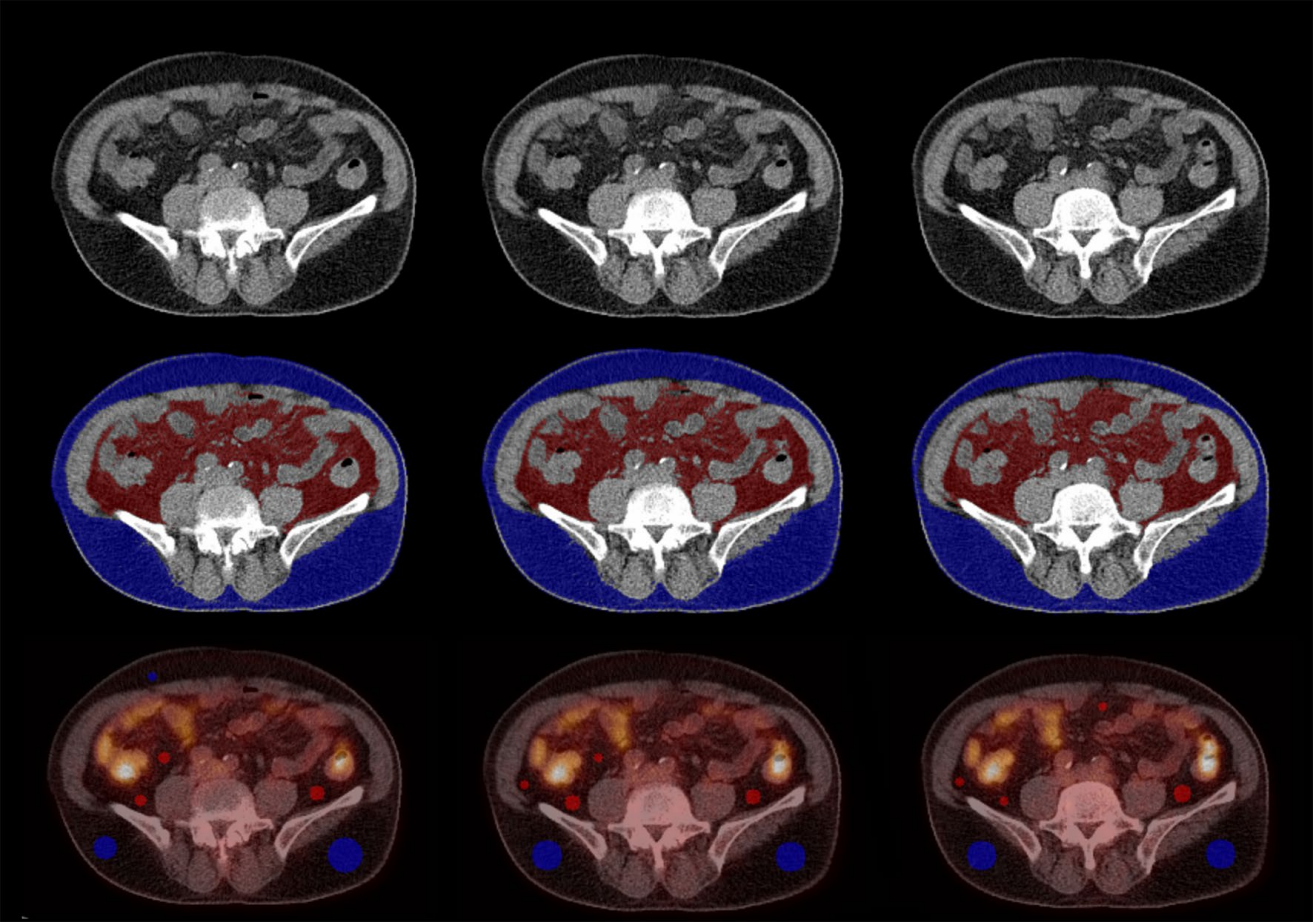
Example of measurement of the volume and standardized uptake value of visceral and subcutaneous tissue from PET/CT images. On the three consecutive trans-axial CT images at the level of L4/5 (top row), the volume of VAT (red) and SAT (blue) were automatically delineated using CT-attenuation range between − 190 and − 30 HU (middle row). On fused PET/CT images, circular ROIs were manually placed on the VAT area while avoiding the spillover from adjacent structures (bottom row). Similarly, circular ROIs were placed on the SAT area (bottom row).

VAT and subcutaneous adipose tissue (SAT) volume were measured on the three consecutive CT images at the L4/5 intervertebral disc level, based on Hounsfield Units (ranging from -190 to -30) (Fig. 1). VAT was defined as intraabdominal fat tissue and SAT as extraperitoneal fat tissue between the skin and muscle. As we suppose that the absolute amount of VAT and SAT may not fully reflect the individual differences in body size, shape, and fat composition, the relative volume was calculated as a proportion of the total adipose tissue volume; for example, relative VAT volume (rVAT) was calculated by dividing the VAT volume by the sum of VAT and SAT volume.

For the measurement of FDG uptake in VAT (VAT-SUV), ROIs (5–10 mm) were placed on the VAT areas of PET images and manually adjusted to avoid spillover from FDG uptake in the bowel, ureter, vessel, and/or muscle, as previously described^13,14^. The average SUV within the ROIs was measured and assigned to the VAT-SUV. For the measurement of FDG uptake in the SAT (SAT-SUV), circular ROIs were drawn on the anterolateral abdominal wall or both buttock areas, whichever was applicable^14^. Then, the average SUV of the ROIs was determined as the SAT-SUV.

The waist circumference (WC) was also measured from the trans-axial CT slice at the level of the L3 transverse process using open-source software named BMI_CT (available at https://sourceforge.net/projects/muscle-fat-area-measurement/). This software automatically calculates the circumference of the body (i.e., WC) from a single CT slice.

### Statistical analysis

Clinicopathological characteristics were analyzed using a variance test where appropriate. The chi-square test or Fisher's exact test was used to compare categorical variables, and the Student *t*-test or Mann–Whitney U test was used to compare continuous variables.

The overall survival (OS) was set as the primary clinical outcome, defined as the time between surgery and mortality of any cause or the last follow-up date. Because rVAT and VAT-SUV were significantly different between men and women, the survival analyses were performed separately. For survival analysis, continuous variables were dichotomized into two groups. The optimal cutoff values for tumor SUV, rVAT, VAT-SUV, and SAT-SUV were determined by the largest χ^2^ on the Mantel-Cox test^15^. The median was used as a cutoff value for WC, and empirical cutoff values were used for other variables. Kaplan–Meier analysis with a log-rank test was used to compare survival differences between the two groups. Univariate and multivariate analyses using Cox proportional hazard regression analysis were performed to identify the independent prognostic factors. Before using the Cox regression model, the proportional hazard assumption for each variable was assessed using the Schoenfeld test, and none of the variables violated the proportional hazards assumption (*p* > 0.05). Variables with P-value < 0.10 on univariate analysis were subjected to the subsequent multivariate analysis. Multivariate stepwise Cox proportional hazard regression models were used to identify the independent prognostic factors for OS.

To investigate the clinical utility of rVAT and VAT-SUV as combined prognostic factors, we divided the patients into four groups: low rVAT and low VAT-SUV (group 1), low rVAT and high VAT-SUV (group 2), high rVAT and low VAT-SUV (group 3), and high rVAT and high VAT-SUV (group 4). Survival differences among the four groups were assessed using Kaplan–Meier analysis with a log-rank test, followed by post hoc tests with Bonferroni multiple comparison correction. The prognostic significance of the combination of rVAT and VAT-SUV was evaluated using the Cox proportional hazard regression model on univariate and multivariate analyses.

Continuous variables are reported as medians with interquartile ranges (IQR), unless specified otherwise. All statistical analyses were conducted in R (version 4.0.3) with 'survival' and 'survminer' packages^16–18^. Statistical significance was set at *p* < 0.05.

## Results

### Patient characteristics

The study population comprised 181 men and 112 women. The median age was 64 years (IQR, 55–72 years), and the body mass index (BMI) was 22.8 kg/m^2^ (20.6–24.9). Left colon cancer was the most common (43.7%), followed by right colon and rectal cancers (28.3% and 28.0%, respectively), and approximately half of the patients were diagnosed with stage I or II CRC (47.4%). After surgery, lymphovascular invasion (LVI) was found in 76 of 293 patients (25.9%), and 189 patients (64.5%) underwent chemotherapy.

Body fat composition and metabolic activity of VAT were significantly different between men and women (Table 1). The relative volume of VAT (i.e., rVAT) was significantly greater in men (0.40 vs. 0.29, *p* < 0.001), that is, the relative volume of SAT was significantly greater in women. The metabolic activity of VAT (i.e., VAT-SUV) was higher in men than in women (0.55 vs. 0.48, *p* = 0.007). However, the SAT-SUV did not differ significantly between men and women. Men were slightly older than women (66 vs. 62, *p* = 0.043), showed higher BMI (23.3 vs. 22.0%, *p* = 0.009), and had a lower histologic grade (G1 and G2; *p* = 0.001). The median WC was greater in men than in women (91.4 vs. 83.7 cm; *p* < 0.001). There were no significant sex differences in other clinicopathological parameters.

**Table 1:** Patients demographics.

	Male (N = 181)	Female (N = 112)	*P*
Age (y)	66.0 (56.0–72.0)	62.0 (52.0–70.5)	0.043
**ASA**			0.933
1	102 (56.35%)	67 (59.82%)	
2	65 (35.91%)	38 (33.93%)	
3 & 4	12 (6.63%)	6 (5.36%)	
No data	2 (1.10%)	1 (0.89%)	
BMI (kg/m^2^)	23.3 (20.9–25.4)	22.0 (19.7–24.4)	0.009
CEA (ng/mL)	3.3 (1.9–6.8)	2.7 (1.5–6.3)	0.323
**Tumor location**			0.117
Rt. Colon	44 (24.3%)	39 (34.8%)	
Lt. Colon	81 (44.8%)	47 (42.0%)	
Rectum	56 (30.9%)	26 (23.2%)	
**Tumor location**			0.117
Rt. Colon	44 (24.3%)	39 (34.8%)	
Lt. Colon	81 (44.8%)	47 (42.0%)	
Rectum	56 (30.9%)	26 (23.2%)	
Tumor size (cm)	4.5 (3.0–6.0)	4.9 (3.5–6.0)	0.183
**Histologic grade**			0.001
G1 & G2	171 (94.5%)	91 (81.2%)	
G3	8 ( 4.4%)	15 (13.4%)	
Etc	2 ( 1.1%)	6 ( 5.4%)	
**LVI**			0.551
Absent	129 (71.3%)	79 (70.5%)	
Present	45 (24.9%)	31 (27.7%)	
No data	7 (3.9%)	2 (1.8%)	
**Stage**			0.554
I & II	83 (45.9%)	56 (50.0%)	
III	76 (42.0%)	40 (35.7%)	
IV	22 (12.2%)	16 (14.3%)	
**MSI**			0.076
MSS	118 (65.2%)	58 (51.8%)	
MSI-Low	10 (5.5%)	13 (11.6%)	
MSI-High	15 (8.3%)	14 (12.5%)	
No data	38 (21.0%)	27 (24.1%)	
**KRAS mutation**			0.780
Absent	42 (23.2%)	29 (25.9%)	
Present	17 ( 9.4%)	12 (10.7%)	
No data	122 (67.4%)	71 (63.4%)	
**Chemotherapy**			0.233
No	59 (32.6%)	45 (40.2%)	
Yes	122 (67.4%)	67 (59.8%)	
Tumor SUV	12.35 (8.61–16.71)	13.00 (9.34–16.69)	0.360
rVAT	0.40 (0.35–0.45)	0.29 (0.23–0.35)	< 0.001
VAT-SUV	0.55 (0.44–0.69)	0.48 (0.41–0.63)	0.007
SAT-SUV	0.33 (0.24–0.43)	0.30 (0.24–0.37)	0.084
WC	83.7 (74.8–90.3)	91.4 (83.8–97.6)	< 0.001

Data are presented as numbers with percentages or median with interquartile ranges.

ASA = American society of anesthesiology, BMI = Body mass index, CEA = Carcinoembryonic antigen, LVI = Lymphovascular invasion, MSI = Microsatellite instability, MSS = Microsatellite Stable, SUV = Standardized uptake value, rVAT = volume ratio of visceral adipose tissue (VAT) to total adipose tissue, SAT = subcutaneous adipose tissue, WC = waist circumference.

### Survival analyses for overall survival

**Figure 2:**
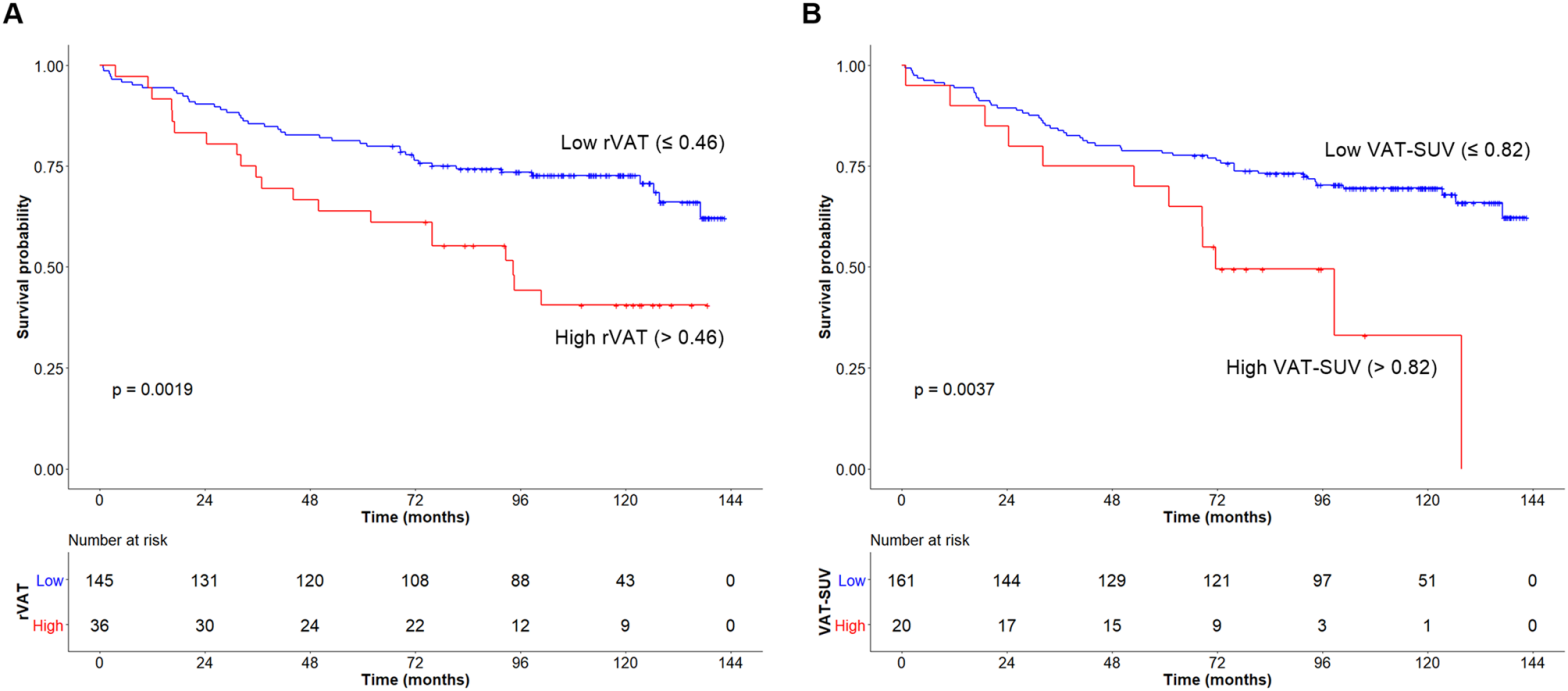
Kaplan–Meier curves of overall survival stratified according to rVAT (**A**) and VAT-SUV (**B**) in males (n = 181).

In male patients, the optimal cutoff values were determined as 8.45 for tumor SUV, 0.46 for rVAT, 0.82 for VAT-SUV, 0.47 for SAT-SUV, and 91.4 cm fo WC. OS was significantly lower in patients with high rVAT than in those with low rVAT (*p* = 0.002), as demonstrated by Kaplan–Meier survival analysis with a log-rank test (Fig. 2A). Similarly, patients with high VAT-SUV showed a worse prognosis than those with low VAT-SUV (*p* = 0.004, Fig. 2B). Univariate analysis showed that age (*p* = 0.004), BMI (*p* = 0.019), LVI (absent vs. present, *p* < 0.001), stage (I and II vs. IV, *p* = 0.002), chemotherapy (*p* = 0.001), rVAT (*p* = 0.002), and VAT-SUV (*p* < 0.001) were associated with OS (Table 2). In the multivariate analysis, LVI (HR 1.92, 95% confidence interval [CI] 1.08–3.40, *p* = 0.025), stage (HR 3.08, 95% CI 1.47–6.48, *p* = 0.003), chemotherapy (HR 0.47, 95% CI 0.27–0.83, *p* = 0.009), rVAT (HR 1.92, 95% CI 1.08–3.42, *p* = 0.026), and VAT-SUV (HR 2.25, 95% CI 1.15–4.41, *p* = 0.018) were independent prognostic factors for OS (Table 2).

**Table 2:** Univariate and multivariate analyses associated with the overall survival in males (n = 181).

	n (%)	Univariate analysis		Multivariate analysis	
		HR (95% CI)	*p*	HR (95% CI)	*p*
**Age**					
< 65	83 (45.9)	Ref		Ref	
≥ 65	98 (54.1)	2.22 (1.30–3.80)	0.004	1.76 (0.98–3.17)	0.061
**BMI**					
< 25	129 (71.3)	Ref			
≥ 25	52 (28.7)	0.46 (0.24–0.88)	0.019		
**CEA**					
< 5	115 (63.5)	Ref			
≥ 5	66 (36.5)	1.64 (1.00–2.69)	0.052		
**Tumor location**					
Rt. Colon	44 (24.3)	Ref			
Lt. Colon	81 (44.8)	0.71 (0.38–1.32)	0.280		
Rectum	56 (30.9)	0.91 (0.48–1.72)	0.767		
**Tumor size**					
< 5	100 (55.2)	Ref			
≥ 5	81 (44.8)	1.22 (0.74–2.00)	0.434		
**Histologic grade**					
G1 & G2	171 (94.5)	Ref			
G3	8 ( 4.4)	2.15 (0.78–5.93)	0.139		
Etc	2 ( 1.1)	1.51 (0.21–10.89)	0.685		
**LVI**					
Absent	129 (71.3)	Ref		Ref	
Present	45 (24.9)	2.56 (1.54–4.28)	< 0.001	1.92 (1.08–3.40)	0.025
No data	7 ( 3.9)	1.06 (0.26–4.40)	0.938	0.85 (0.20–3.66)	0.831
**No. of retrieved LNs**					
< 12	35 (19.3)	Ref			
≥ 12	146 (80.7)	0.71 (0.40–1.25)	0.238		
**Stage**					
I & II	83 (45.9)	Ref		Ref	
III	76 (42.0)	1.39 (0.80–2.41)	0.244	1.60 (0.84–3.04)	0.153
IV	22 (12.2)	3.03 (1.50–6.13)	0.002	3.08 (1.47–6.48)	0.003
**KRAS mutation**					
Absent	42 (23.2)	Ref			
Present	17 ( 9.4)	1.11 (0.42–2.93)	0.829		
No data	122 (67.4)	1.00 (0.53–1.86)	0.990		
**Chemotherapy**					
No	59 (32.6)	Ref		Ref	
Yes	122 (67.4)	0.43 (0.26–0.70)	0.001	0.47 (0.27–0.83)	0.009
**Tumor SUV**					
Low	78 (43.1)	Ref			
High	103 (56.9)	0.74 (0.45–1.21)	0.227		
**WC**					
Low	161 (89.0)	Ref			
High	20 (11.0)	0.79 (0.48–1.29)	0.342		
**rVAT**					
Low	90 (49.7)	Ref		Ref	
High	91 (50.3)	2.28 (1.34–3.88)	0.002	1.92 (1.08–3.42)	0.026
**VAT-SUV**					
Low	145 (80.1)	Ref		Ref	
High	36 (19.9)	2.49 (1.32–4.69)	0.005	2.25 (1.15–4.41)	0.018

ASA = American society of anesthesiology, BMI = Body mass index, CEA = Carcinoembryonic antigen, LN = Lymph node, LVI = Lymphovascular invasion, SUV = Standardized uptake value, rVAT = volume ratio of visceral adipose tissue (VAT) to total adipose tissue, WC = waist circumference.

**Figure 3:**
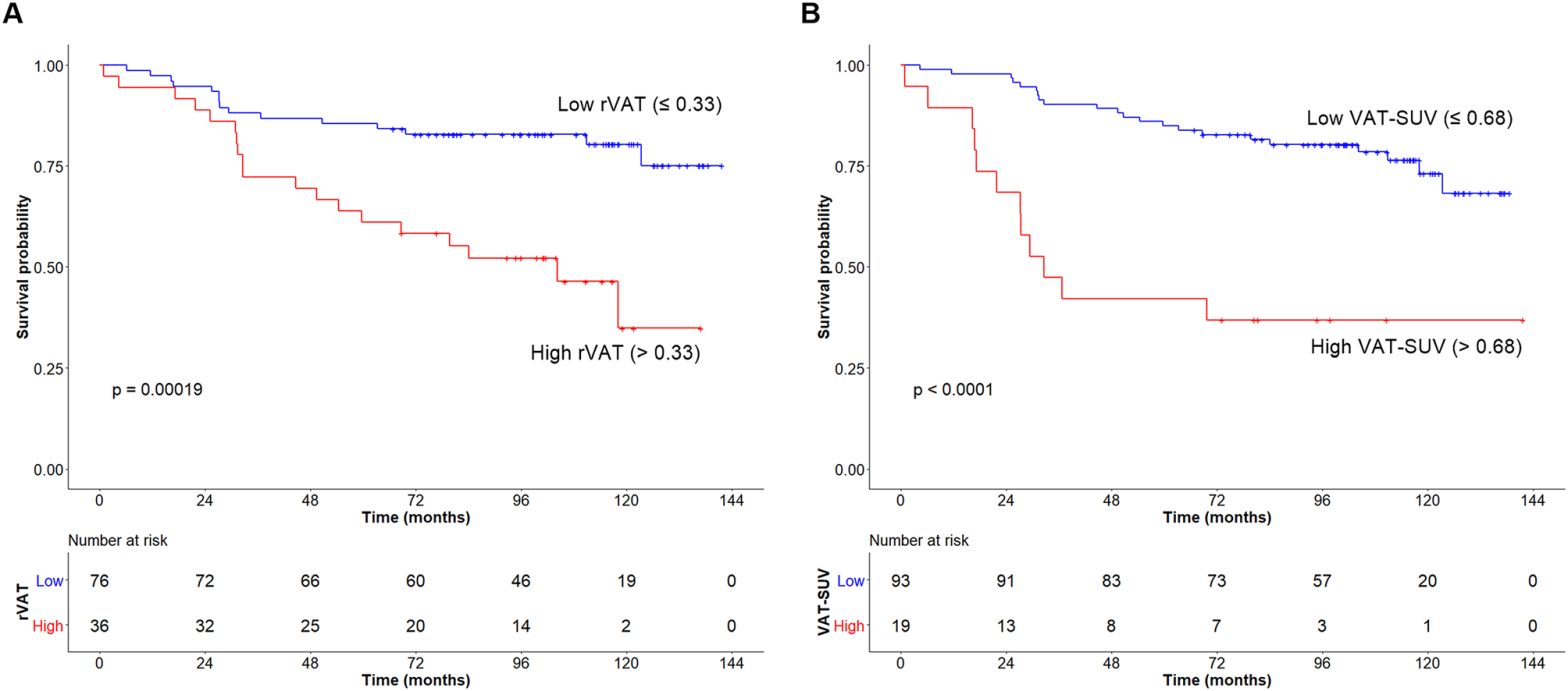
Kaplan–Meier curves of overall survival stratified according to rVAT (**A**) and VAT-SUV (**B**) in females (n = 112).

In female patients, the optimal cutoff values were determined as 10.76 for tumor SUV, 0.33 for rVAT, 0.68 for VAT-SUV, 0.39 for SAT-SUV, and 83.7 cm for WC. OS was significantly shorter in patients with high rVAT than in those with low rVAT (*p* < 0.001, Fig. 3A). Patients with high VAT-SUV showed a worse prognosis than those with low VAT-SUV (*p* < 0.001, Fig. 3B). Univariate analysis revealed that CEA (*p* = 0.030), LVI (absent vs. present, *p* = 0.024), stage (I and II vs. IV, *p* < 0.001), rVAT (*p* < 0.001), and VAT-SUV (*p* < 0.001) were associated with OS. Chemotherapy (no vs. yes) and stage (I & II vs. III) showed a marginal significance of *p* = 0.050 and 0.051, respectively. In the multivariate analysis, stage (I &II vs. III, HR 4.24, 95% CI 1.51–11.89, *p* = 0.006; I & II vs. IV, HR 14.21, 95% CI 5.52–36.59, *p* < 0.001), chemotherapy (HR 0.38, 95% CI 0.18–0.84, *p* = 0.017), rVAT (HR 2.96, 95% CI 1.38–6.34, *p* = 0.005), and VAT-SUV (HR 4.45, 95% CI 2.03–9.75, *p* < 0.001) were determined as independent prognostic factors for OS (Table 3).

**Table 3:** Univariate and multivariate analyses in females (n = 112).

	n (%)	Univariate analysis		Multivariate analysis	
		HR (95% CI)	*p*	HR (95% CI)	*p*
**Age**					
< 65	67 (59.8)	Ref			
≥ 65	45 (40.2)	1.38 (0.7–2.71)	0.350		
**BMI**					
< 25	92 (82.1)	Ref			
≥ 25	20 (17.9)	0.71 (0.27–1.83)	0.476		
**CEA**					
< 5	81 (72.3)	Ref			
≥ 5	31 (27.7)	2.14 (1.08–4.26)	0.030		
**Tumor location**					
Rt. Colon	39 (34.8)	Ref			
Lt. Colon	47 (42.0)	1.33 (0.62–2.85)	0.461		
Rectum	26 (23.2)	0.75 (0.28–2.02)	0.565		
**Tumor size**					
< 5	56 (50.0)	Ref			
≥ 5	56 (50.0)	1.56 (0.79–3.08)	0.205		
**Histologic grade**					
G1 & G2	91 (81.2)	Ref			
G3	15 (13.4)	0.17 (0.02–1.24)	0.080		
Etc	6 ( 5.4)	0.96 (0.23–4.02)	0.956		
**LVI**					
Absent	79 (70.5)	Ref			
Present	31 (27.7)	2.20 (1.11–4.36)	0.024		
No data	2 ( 1.8)		0.996		
**No. of retrieved LNs**					
< 12	18 (16.1)	Ref			
≥ 12	94 (83.9)	0.80 (0.35–1.85)	0.604		
**Stage**					
I & II	56 (50.0)	Ref		Ref	
III	40 (35.7)	2.51 (1.00–6.28)	0.050	4.24 (1.51–11.89)	0.006
IV	16 (14.3)	17.77 (7.09–44.51)	0.000	14.21 (5.52–36.59)	< 0.001
**KRAS mutation**					
Absent	29 (25.9)	Ref			
Present	12 (10.7)	0.4 (0.05–3.28)	0.389		
No data	71 (63.4)	1.74 (0.71–4.27)	0.227		
**Chemotherapy**					
No	45 (40.2)	Ref		Ref	
Yes	67 (59.8)	0.51 (0.26–1.00)	0.051	0.38 (0.18–0.84)	0.017
**Tumor SUV**					
Low	42 (37.5)	Ref			
High	70 (62.5)	0.6 (0.31–1.18)	0.139		
**WC**					
Low	56 (50.0)	Ref			
High	56 (50.0)	3.43 (1.73–6.82)	< 0.001		
**rVAT**					
Low	76 (67.9)	Ref		Ref	
High	36 (32.1)	3.43 (1.73–6.82)	< 0.001	2.96 (1.38–6.34)	0.005
**VAT-SUV**					
Low	93 (83.0)	Ref		Ref	
High	19 (17.0)	4.77 (2.33–9.76)	< 0.001	4.45 (2.03–9.75)	< 0.001

ASA = American society of anesthesiology, BMI = Body mass index, CEA = Carcinoembryonic antigen, LN = Lymph node, LVI = Lymphovascular invasion, SUV = Standardized uptake value, rVAT = volume ratio of visceral adipose tissue (VAT) to total adipose tissue, WC = waist circumference.

The Schoenfeld test indicated no violation of the proportional hazards assumption in the multivariate Cox models for male (*p* = 0.189) and female (*p* = 0.230) patients.

### Combination of rVAT and VAT-SUV

**Figure 4:**
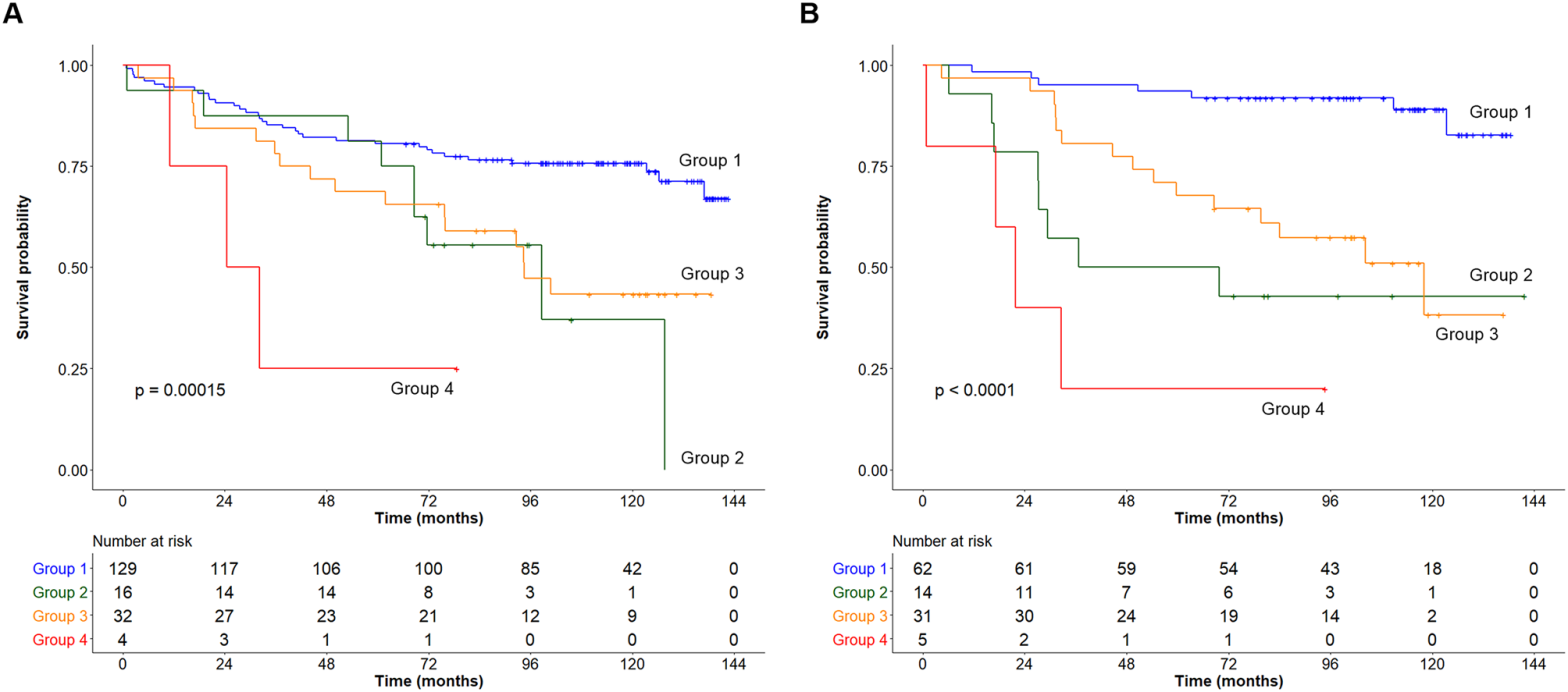
Overall survival estimation in the combination of rVAT and VAT-SUV in males (**A**) and females (**B**). Group 1: low rVAT and low VAT-SUV; Group 2: low rVAT and high VAT-SUV; Group 3: high rVAT and low VAT-SUV; Group 4: high rVAT and high VAT-SUV.

Kaplan–Meier survival analyses demonstrated a significant difference in survival outcomes among the four groups in both male (Fig. 4A) and female patients (Fig. 4B) (*p* < 0.001). In male patients, post hoc tests with multiple comparison adjustment showed that group 1 (low rVAT and low VAT-SUV) had a better survival outcome than group 3 (high rVAT and low VAT-SUV) and group 4 (high rVAT and high VAT-SUV) (*p* < 0.05). However, there were no significant differences between the other groups. Multivariate analysis of the prognostic model using the combination of rVAT and VAT-SUV indicated that the four-group variable was an independent prognostic factor for predicting OS in men (group 1 vs. group 4, HR 7.16, 95% CI 2.06–24.88, *p* = 0.002), along with LVI, stage, and chemotherapy (Table 4). In contrast, in women, group 1 showed significantly longer survival than group 2, group 3, and group 4 (*p* < 0.05, all pair-wise comparisons), and group 3 also showed significantly longer survival than group 4 (*p* = 0.03). In multivariate analysis, the four-group variable was significantly associated with OS, independent of stage and chemotherapy (1 vs. 2, HR 7.16, *p* = 0.003; 1 vs. 3, HR 3.30, *p* = 0.018; 1 vs. 4, HR 12.68, p < 0.001) (Table 4). Notably, among female patients, the combination of rVAT and VAT-SUV could identify those with the best survival outcome (i.e., group 1) from the other three individual groups. According to the Schoenfeld test, there was no violation of the proportional hazard assumption in the multivariate Cox models for male (*p* = 0.094) and female (*p* = 0.288) patients.

**Table 4:** Multivariate model using the combination of rVAT and VAT_SUV.

	Male (n = 181)			Female (n = 112)		
	n (%)	HR (95% CI)	*p*	n (%)	HR (95% CI)	*p*
**Age**						
< 65	83 (45.9)	Ref				
≥ 65	98 (54.1)	1.77 (0.99–3.18)	0.054			
**LVI**						
Absent	129 (71.3)	Ref				
Present	45 (24.9)	1.92 (1.08–3.43)	0.026			
No data	7 ( 3.9)	0.84 (0.19–3.59)	0.811			
**Stage**						
I & II	83 (45.9)	Ref		56 (50.0)	Ref	
III	76 (42.0)	1.57 (0.82–3.01)	0.175	40 (35.7)	4.13 (1.46–11.74)	0.008
IV	22 (12.2)	3.16 (1.49–6.71)	0.003	16 (14.3)	13.69 (5.22–35.89)	< 0.001
**Chemotherapy**						
No	59 (32.6)	Ref		45 (40.2)	Ref	
Yes	122 (67.4)	0.45 (0.25–0.79)	0.006	67 (59.8)	0.40 (0.18–0.88)	0.023
**rVAT & VAT-SUV**						
Low-Low	129 (71.3)	Ref		62 (55.4)	Ref	
Low–High	16 ( 8.8)	1.85 (0.84–4.06)	0.125	14 (12.5)	5.06 (1.73–14.82)	0.003
High-Low	32 (17.7)	1.71 (0.91–3.18)	0.094	31 (27.7)	3.30 (1.23–8.83)	0.018
High-High	4 ( 2.2)	7.16 (2.06–24.88)	0.002	5 ( 4.5)	12.68 (3.38–47.55)	< 0.001

BMI = Body mass index, CEA = Carcinoembryonic antigen, LVI = Lymphovascular invasion, rVAT = volume ratio of visceral adipose tissue (VAT) to total adipose tissue, SUV = Standardized uptake value.

## Discussion

This study demonstrated that higher glucose uptake in visceral fat and higher relative volume of visceral fat were strongly associated with poor prognosis in patients with CRC. The combination of rVAT and VAT-SUV has been proven to be an independent prognostic factor. However, the discriminatory impact of the combination of these two parameters on OS was more prominent in women than in men. This study highlighted the clinical relevance of adding glucose uptake of visceral fat as a potential predictor of OS, especially in women.

A growing body of evidence has evaluated the clinical significance of visceral fat area (VFA); however, contradictory results have been observed. Ebadi et al. reported that no difference was observed between high and low visceral fat index (VFI) in patients with various types of cancers (HR 1.13, *p* = 0.08)^6^. However, VAT > 44 cm^2^ was analyzed as an independent prognostic factor (HR 2.61, 95% CI 1.15–5.92, *p* = 0.021) in patients with metastatic CRC^19^. Although our study analyzed the volume of adiposity as a potential imaging biomarker, several issues should be emphasized. First, measuring the volume of visceral or subcutaneous fat area in PET/CT employed in this study was not identical to the previous approach, which used a slice of CT image at the L3 level. Therefore, the absolute volume of VAT in our study was different from the adiposity area measured with L3 level-based calculation. Second, most of the previous studies used absolute values, such as VFA or height-normalized VFA, which is usually denoted as VFI. It was suggested that the absolute quantity of visceral fat could not control for increased concomitant subcutaneous fat, and considering relative VAT could compensate for this limitation^11^. We also calculated rVAT and used it as a quantitative biomarker. Our results showed that large rVAT was related to poor prognosis in men and women, although the optimal cutoff was somewhat different by sex. The concept of the relative volume of visceral fat can be applied equally to studies measuring VFA using an L3 level-based calculation. Because the absolute cutoff value of VFA may inevitably vary among different ethnicities, using rVAT as a prognosticator can have more benefit in general usage, although additional verification is required for this hypothesis.

Although the prognostic significance of glucose uptake of visceral fat has been previously proven in various types of cancers, including CRC, none of these studies evaluated the different impacts according to sex^7–9^. The present study has strength in investigating the prognostic impact of metabolic activity of the VAT by sex. Tahara et al. reported that target to background ratio (TBR) of VAT showed no significant difference between sexes (mean SUV ± standard deviation, 0.5 ± 0.09 in men vs. 0.49 ± 0.12 in women, *p* = 0.551) in 251 (172 men) healthy individuals who underwent PET/CT for risk-screening tests for cardiovascular disease^20^. In contrast, Kwon et al. reported that the mean SUV of VAT is significantly higher in women than in men (0.63 ± 0.10 in women vs. 0.51 ± 0.10 in men, *p* < 0.001) among 232 consecutive subjects who were participants of a health screening program^21^. However, the included number of women in that study was only 12, which would be too small to have adequate statistical power. In our study, we found that not only the relative volume of VAT but also median visceral fat SUV uptake showed sex dependency, and men showed higher median rVAT and median VAT-SUV than women (0.55 [IQR 0.44–0.69] in men vs. 0.48 [IQR 0.41–0.63] in women, *p* = 0.007). Therefore, we defined the cutoff values of rVAT and VAT-SUV differently according to sex, although the absolute difference between sexes was low, especially for VAT-SUV. In multivariate analysis, rVAT and VAT-SUV were identified as independent prognostic factors in both men and women. When rVAT and VAT-SUV were combined, survival differences among the four groups were prominent in female patients, whereas differences were evident only between patients with low rVAT and low VAT-SUV versus high rVAT and high VAT-SUV in male patients. Considering that only four patients were allocated to the subgroup with high rVAT with high VAT-SUV, the four-group stratification might have limited clinical value for risk stratification in male patients. Taken together, these results indicate that VAT-SUV could have an incremental effect in stratifying patients' survival, especially in female patients. Nguyen et al. investigated the sex difference in rVAT and demonstrated that women with rVAT greater than 30.9% showed an increased risk of death in clear cell renal cell carcinoma, whereas this dichotomization for survival was not possible in male patients. Furthermore, the combination of rVAT and glycolytic gene expression could separate patients with exceptionally good prognosis, especially in women^11^. Although this prior observation could infer that the fundamental response of adiposity metabolic activity showed sex differences with respect to survival, the reason for this difference cannot be explained by our analysis, and this would be an interesting subject for further research.

We believe that our findings could enhance patient stratification, even in preoperative decision-making. The impact of neoadjuvant chemotherapy on locally advanced CRC is being increasingly investigated. A recent randomized trial reported no additional benefit for perioperative chemotherapy in patients who were clinically diagnosed as high-risk, such as T3, T4, and/or N2 colon cancer on preoperative CT scan. Nevertheless, identifying a subgroup of patients who could benefit from this strategy is warranted^22^. In this respect, preoperative imaging biomarkers that can stratify high-risk groups are essential, and the clinical usefulness of visceral fat-derived classification needs to be investigated in the future.

This study had some limitations. This was a retrospective single-center study; thus, selection bias was inevitable. In addition, cutoff values of rVAT and VAT-SUV were derived and evaluated in the same patient population. Further validation is required by a multicenter study involving a large number of patients. Finally, we did not consider recurrence-associated outcomes, such as progression-free survival.

In the present study, we demonstrated that rVAT and VAT-SUV could have prognostic importance in a sex-specific manner and that the combination of rVAT and VAT-SUV could discriminate patient survival in more detail, especially in female patients. Thus, we propose a new stratification system that integrates the sex of the patient, quantity, and glucose uptake of visceral fat in patients with CRC.

## Data Availability

The datasets analyzed in this study are not publicly available owing to ethical and privacy restrictions; they are available from the corresponding author on reasonable request.
